# Postoperative spinal cord infarction on a gravid woman with suspected IV drug use: a case report

**DOI:** 10.1186/s12884-022-05157-1

**Published:** 2022-12-07

**Authors:** Janine N. Baldino, Johnathon Recknagel, A. Jenna Beckham

**Affiliations:** 1 Department of Obstetrics and Gynecology, University of North Carolina at Chapel Hill, Chapel Hill, NC USA; 2 WakeMed Health and Hospitals, Raleigh, NC USA

**Keywords:** IV drug use in pregnancy, Back pain in pregnancy, Spinal cord infarction, Risk of spinal cord injury in pregnancy, Infection in pregnancy

## Abstract

**Background:**

Back pain is common in the gravid population and spinal cord infarction (SCI) or chronic osteomyelitis are exceptionally rare underlying causes of back pain in this population. No case report to date has described this unexpected adverse event in a gravid woman with suspected history of IV drug use (IVDU). This diagnosis could potentially become more common with increasing rates of IVDU, and increased education could result in sooner recognition.

**Case:**

A 38 year old G9P0171 at 24 weeks gestation with a complex past medical history, and a suspected history of IVDU, presented repeatedly with back pain. Following cesarean delivery at 36w2d, she developed signs and symptoms of an anterior spinal artery syndrome (ASAS) and had evidence of chronic osteomyelitis at T9-T10 on imaging. This required emergent decompressive laminectomy and ultimately resulted in paraplegia.

**Conclusion:**

This case highlights the difficulties in recognizing all SCI risk factors pre-operatively and the importance of investigating back pain in pregnant patients with a suspected history of IVDU. We believe this patient’s chronic infection put her at an increased risk for SCI that was possibly compounded by the anatomical changes from its chronicity, possibly occurring in combination with several other precipitating causes of hypoperfusion. We hope this case report highlights the modern necessity to include a history, or suspected history, of IVDU as a red flag to initiate imaging in pregnant patients with acute, persistent, or unresolved back pain.

Spinal cord infarctions (SCI) are rare occurrences, even in the general population. This rarity makes it difficult to estimate the true incidence, however several estimates put the incidence at 0.3–1.2% of all strokes [1–3]. Incidence during pregnancy is increasingly difficult to estimate as most are reported through single case reports and most cannot identify a precise cause [2, 4, 5]. Simply, SCI happens after a detrimental decrease in perfusion pressure or direct interference with blood supply. These outcomes can happen after a wide range of events. Hypotension, surgery, vasculitis, arterial injury or compromise (through infection, occlusion, vasospasm or constriction), anatomical abnormalities, trauma, coagulation abnormalities, thrombosis, or a combination of any have all been hypothesized correlated causes [6–8]**.** This means those with atherosclerotic disease, diabetes, or iatrogenic vertebral injury are at risk [1]. Infection has become a risk factor of particular interest, especially for pregnant women with a history of intravenous drug use disorders [9–12]. With drug use on the rise in people aged 12 and older, an abundance of studies and reports are demonstrating the serious effects of injection drugs such as bacteremia, infective endocarditis, spinal infections, and abscess. The pregnant population is not immune to these devastating sequela [9–13].

In this case report, we walk through a case of SCI after repeat cesarean section in a patient who was found to have chronic osteomyelitis postoperatively, suspected to be caused by hematogenous spread from drug injection sites. No reports to date have been published regarding SCI in an obstetrical patient with chronic infection likely from intravenous drug use (IVDU). Specifically, none address the possible increase in risk for this in the gravid population with a history of substance use disorders. Through this case, we review the complex pathophysiology of several main risk factors involved in this case, with a focus on IVDU and bring awareness to IVDU as a new red flag that should prompt further investigation in gravid women with back pain.

## Case report

A 37-year-old G9P0171 (seven spontaneous abortions reported) with a history of chronic hypertension, type 2 diabetes mellitus, chronic kidney disease, neuropathy with Charcot foot, class III obesity with a BMI 52, and prior preterm delivery for superimposed preeclampsia transferred her care to our high-risk practice at 24w2d. During her initial prenatal visit, she also disclosed a history of multiple MRSA containing skin abscesses on her chest that she reported had originated from spider bites.

Prior to transferring her care, she first presented to the Emergency Department with shooting left upper back and flank pain at 18 weeks. At that time at the outside institution, a CT scan without contrast was ordered to evaluate for nephrolithiasis, but the abdomen visualized inferiorly from the diaphragm was largely unremarkable. She presented to the Emergency Department six more times from 18 through 34 weeks gestation with complaints of ongoing pain, occasionally describing the pain shooting down her legs and causing lower extremity numbness. Throughout her visits no motor or sensory deficits were elicited on exam, and she was repeatedly afebrile with no pain on spine palpation. She was treated with acetaminophen and cyclobenzaprine for possible paraspinal muscle spasm. During one of these visits at 33 weeks a urine drug screen was obtained by the triage provider due to their concerns about the patient’s poor compliance with prenatal care appointments, as she had not presented to scheduled visits for over eight weeks. This screen resulted positive for fentanyl and was negative for all other substances included in the basic toxicology screen. She adamantly denied any opioid use, presenting a diagnostic challenge, and requested repeat testing the following day as well as five days later. Both subsequent tests were positive. The patient continued to deny any opioid use, however the placement and number of infected skin lesions on her chest raised suspicion for injection drug use. At a much later date, she disclosed she had been a restrained passenger in a motor vehicle collision during the first trimester of her pregnancy and stated this was the first time she noticed the back pain.

At 36w1d, she was directed to the Obstetrics Emergency Department for evaluation after a biophysical profile score of 2/8 and amniotic fluid index of 0.7 was noted during routine antenatal testing. At this time, she also reported that her back pain had been significantly exacerbated by a fall en route to the hospital. Due to reactive non-stress test upon arrival, decision was made for observation until delivery via repeat cesarean section at the next available scheduled OR slot. Anesthesia attempted to place a combined spinal and epidural block at L2-L3, as was the standard of care for obese patients. Following the initial spinal dose, placement of the epidural catheter was abandoned after the patient experienced pressure and pain with local anesthetic infusion. The procedure continued under single-shot spinal anesthesia. Her mean arterial pressure (MAP) dropped to 75 mmHg for a few minutes following the spinal, and then corrected to her baseline MAP of 115–125 mmHg with fluid resuscitation prior to the procedure. The repeat cesarean section was then completed and was overall uncomplicated. Final quantitative blood loss was 910 mL. Her neonate had 1 and 5 min APGAR scores of 3 and 7, respectively, initially requiring some positive pressure ventilation but with an overall uncomplicated neonatal course.

While in the post anesthesia care unit, she developed severe range blood pressures (systolic pressures persisting in the 170 s over fifteen minutes apart) requiring acute treatment with labetalol and hydralazine. Complete blood count, complete metabolic panel, lactate dehydrogenase, urine protein to creatine ratio (U P:C) were obtained which were unremarkable aside from a significant increase from baseline in her U P:C. Superimposed pre-eclampsia with severe features was diagnosed and intravenous magnesium sulfate was initiated for seizure prophylaxis and continued over the next 24 h. Her MAP nadired at 68 mmHg during this 24 h period, and she did not require further acute treatment of severe range blood pressures. Throughout treatment she continued to complain of back pain at a similar severity to the antepartum period. Since reflexes in the bilateral lower extremities were poorly elicited and this was thought to be her baseline, reflexes in her upper extremities were used for her serial examinations while receiving magnesium. She was noted to have full voluntary motor function in all four extremities during this time.

**Fig. 1:**
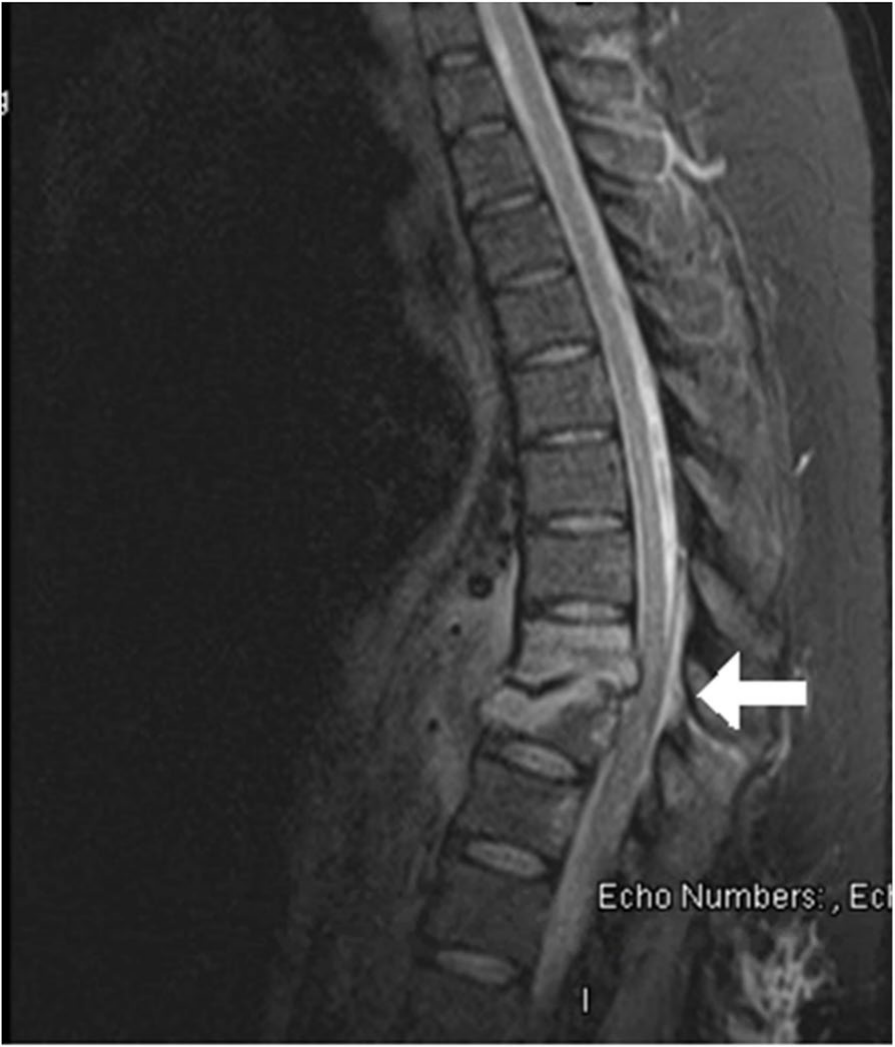
STIR Sequence sagittal image with T9-10 lesion noted with white arrow

**Fig. 2:**
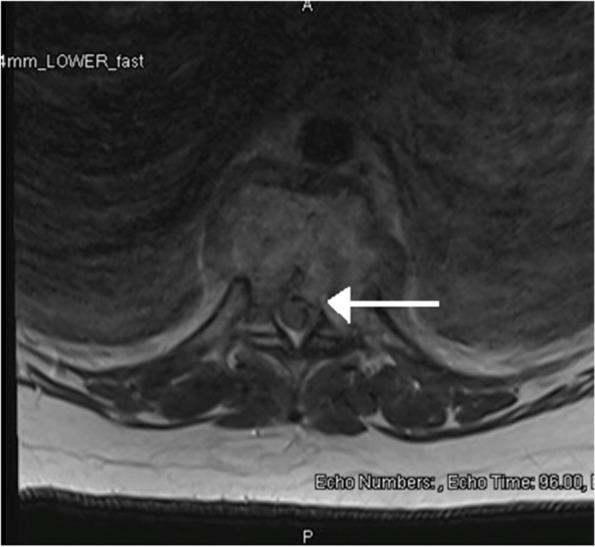
T2 Weighted axial image with canal stenosis noted with white arrow

**Fig. 3:**
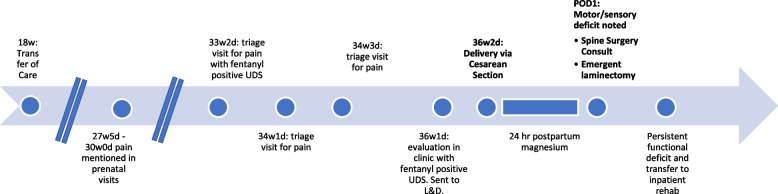
Timeline of Clinical Events

On post-operative day one, after completion of magnesium treatment, the patient complained of inability to move her legs. Physical examination demonstrated impaired pain and light touch sensation below the umbilicus, no voluntary strength in bilateral lower extremities, no Babinski or ankle jerk reflex, and the patient was unable to void after Foley catheter removal. MRI of the spine showed discitis osteomyelitis at T9-T10 with phlegmonous change in the soft tissues anterior to the vertebral column. This phlegmonous area extended into the ventral and dorsal epidural space resulting in severe spinal cord narrowing at T9-T10. There was also focal kyphosis in this region, indicating chronicity of some of these changes. MRI images are displayed in Figs. 1 and 2. Orthopedic surgery was consulted and the patient was taken for emergent T9-T10 laminectomy, wound debridement, and T7-T12 fusion for suspected spinal cord infarction secondary to arterial disruption in the setting of infection. Intra-operatively, a “thick phlegmon” was noted overlying the dura which was dissected away to decompress the cord. She received 4 units of packed red blood cells and 2 units of fresh frozen plasma intraoperatively due to neurogenic shock. Cultures collected during surgery grew Methicillin-sensitive Staphylococcus aureus. Postoperatively, she had persistent bilateral lower extremity motor and sensory deficit and was admitted to inpatient rehabilitation. Her lower extremity function and sensation improved over months of physical therapy. As of four months following the initial injury, the patient had been discharged from the hospital and was undergoing home physical therapy. She was not ambulatory but could stand with support equipment. Please refer to Fig. 3 for a visual of the clinical timeline.

## Discussion

SCI is a rare spinal cord injury and often has many contributing factors to its etiology. Catching signs and symptoms early can be particularly challenging when masked postoperatively by anesthesia or pain medication. This patient exemplifies many etiological factors at play, especially in an obstetrical patient. Her symptomatology of bilateral lower extremity motor deficit, voiding difficulty, areflexia, and loss of pain and touch sensation below her umbilicus along with evidence of SCI on MRI fulfill almost all of the diagnostic criteria for an anterior spinal artery syndrome (ASAS) [6, 14] from a SCI at the level of T10. Sparing of proprioception and vibratory sense would fulfill the criteria completely, however she lacked these on physical exam. While ASAS was highest on the differential, whatever the inciting injury was had progressed to full compression of all spinal tracts beginning at T10.

Due to the coinciding injured spinal region and timing prior to onset of ASAS symptoms in this patient, administration of neuraxial anesthesia initially seems to be a chief precipitating cause of this injury. However, this is a very rare cause of ASAS in the obstetrical population. It is difficult to accurately estimate exactly how rare this occurrence is, however a retrospective study of more than half a million obstetrical patients in the United Kingdom showed only one patient who developed ASAS after neuraxial anesthesia [15]. An updated study looking at serious complications in more than 250,000 obstetrical anesthesia patients had too few complications to identify true risk factors and SCI was not a reported outcome [16].

Peri- and intra-operative hypotension has recently gained more attention as contributing to SCI, especially midthoracic ASAS since the mid thoracic cord is considered the ‘critical watershed area’ [14]. The anterior spinal artery of the thoracic cord receives blood supply from radicular arteries, particularly the radicular artery of Adamkiewicz. Unfortunately, the mid thoracic region does not receive as many of these radicular branches and therefore has a heightened vulnerability to hypoperfusion [7, 15, 17]. Cesarean delivery alone is not considered a procedure at risk for this kind of spinal cord ischemia [15] and it is unlikely her hypotension alone, which was not particularly remarkable, was a large contributing cause of SCI in this patient.

Most notable in this patient is her spinal cord narrowing and kyphosis from chronic infection. This stenotic obstruction from vascular changes due to a chronic infection was likely disrupting normal blood flow to varying degrees and greatly increased her risk of SCI. While pre-existing infection and spinal abnormalities are contraindications to neuraxial anesthesia, they must be identified first. This patient ultimately denied all opioid use, but multiple drug screens and MRSA cellulitis strongly suggested otherwise. Illicit drug use, including opioids, in the reproductive aged population is still a public health concern in the United States. While the national survey on drug use and health performed by the Substance Abuse and Mental Health Services Administration in 2019 reports heroin use in the past 30 days by pregnant women has gone from 1.4% in 2017 to 0.4% in 2019 [18], the age-adjusted rate of total overdose deaths increased by 4% from 2018 to 2019 [19]. A retrospective analysis of the National Survey on Drug Use and Health from 2005–2014 reported 5.1% of pregnant women in the U.S. having used a nonmedical opioid in the past year and 0.9% in the past month [20]. 20% of pregnant women aged 15–17 report current illicit drug use and 5% of those pregnant women aged 15–44. While the incidence of intravenous drug use in pregnancy in the United States is unknown, neonatal abstinence syndrome has increased from 1.2 to 3.39 per 1,000 births from 2000–2009 [21]. It can be assumed that this rise in total encompasses a rise in IVDU. These are also likely a underestimations due to underreporting. Based on this data, it is no surprise we encounter the downstream effects of widespread opioid use, especially infection. One retrospective case series looking at hospitalized pregnant patients with S. aureus bacteremia found 85% of those patients reported active injection drug use and only 19% presented with fever [9]. These infections can hematogenously seed elsewhere and the spine is a common place for this [11, 12]. Given this patient’s significant comorbidities of obesity, diabetes and recurrent MRSA infections, she was at higher risk than the average population to develop infectious complications. Diagnosis of these secondary spinal infections is often delayed due to insidious onset and lack of symptoms other than back pain. A study of 164 patients with spinal infections, where 102 had a history of IVDU, showed that the majority had back pain as their only symptom [11]. Absence of fever or abnormal leukocytes is not uncommon in presentations of spinal infections [11–13]. This can further complicate the decision on when to image these patients. Imaging every pregnant woman with back pain or history of IVDU puts undue stress on the healthcare system and patients.

A retrospective study aimed at determining the necessity to MRI every case of back pain in patients with a history of IVDU demonstrated its use *is* justified in patients with vague presentations of spinal infection such as non-specific lab markers, absent fever, and ambiguous back pain [13]. Of the 167 patients with a history of IVDU and acute back pain, MRI detected evidence of spinal infections in 40% of them. Given this, a known, or strongly suspected, history of IVDU coupled with acute, persistent, or changing back pain should increase suspicion for spinal infection enough to warrant MRI workup. In regards to this case, the patient denied a history of IV drug use, although her signs and symptoms strongly suggested there may have been, and no satisfactory alternative explanation was found for these results.

This case report, while novel, is somewhat limited by the inconclusive investigation into the etiology of her osteomyelitis. Multiple positive fentanyl screens suggest undisclosed drug use, but this was not firmly established. There was also difficulty in establishing the link between the cesarean delivery and the exacerbation of her condition—while the timing suggests that the circumstances surrounding her cesarean delivery precipitated this injury it could merely be an inevitable consequence of her pre-existing spinal infection.

This case highlights the difficulties in recognizing all SCI risk factors pre-operatively and the importance of investigating back pain in pregnant patients with a suspected history of IVDU. We believe this patient’s chronic infection put her at an increased risk for SCI that was possibly compounded by the anatomical changes from its chronicity, possibly occurring in combination with several other precipitating causes of hypoperfusion. We hope this case report highlights the modern necessity to include a history, or suspected history, of IVDU as a red flag to initiate imaging in pregnant patients with acute, persistent, or unresolved back pain.

## Data Availability

Not applicable.

## Abbreviations

IVDU: IV Drug use
SCI: Spinal Cord Infarctions
MAP: Mean Arterial Pressure
ASAS: Anterior Spinal Artery Syndrome
U P:C: Urine Protein to Creatinine Ratio
